# Investigating the Performance of Generative Adversarial Networks for Prostate Tissue Detection and Segmentation

**DOI:** 10.3390/jimaging6090083

**Published:** 2020-08-24

**Authors:** Ufuk Cem Birbiri, Azam Hamidinekoo, Amélie Grall, Paul Malcolm, Reyer Zwiggelaar

**Affiliations:** 1 Department of Computer Engineering, Middle East Technical University, Ankara 06800, Turkey; cem.birbiri@metu.edu.tr; 2 Division of Molecular Pathology, Institute of Cancer Research (ICR), London SM2 5NG, UK; azam.nekoo@icr.ac.uk; 3 Probayes, 38330 Montbonnot, France; ameliegrall5@gmail.com; 4 Department of Radiology, Norfolk & Norwich University Hospital, Norwich NR4 7UY, UK; paul.malcolm@nnuh.nhs.uk; 5 Department of Computer Science, Aberystwyth University, Aberystwyth SY23 3DB, UK

**Keywords:** prostate MRI, computer aided diagnosis, segmentation, detection, generative adversarial network

## Abstract

The manual delineation of region of interest (RoI) in 3D magnetic resonance imaging (MRI) of the prostate is time-consuming and subjective. Correct identification of prostate tissue is helpful to define a precise RoI to be used in CAD systems in clinical practice during diagnostic imaging, radiotherapy and monitoring the progress of disease. Conditional GAN (cGAN), cycleGAN and U-Net models and their performances were studied for the detection and segmentation of prostate tissue in 3D multi-parametric MRI scans. These models were trained and evaluated on MRI data from 40 patients with biopsy-proven prostate cancer. Due to the limited amount of available training data, three augmentation schemes were proposed to artificially increase the training samples. These models were tested on a clinical dataset annotated for this study and on a public dataset (PROMISE12). The cGAN model outperformed the U-Net and cycleGAN predictions owing to the inclusion of paired image supervision. Based on our quantitative results, cGAN gained a Dice score of 0.78 and 0.75 on the private and the PROMISE12 public datasets, respectively.

## 1. Introduction

Prostate cancer is the most common type of cancer in men. In 2017, there were nearly 48,500 new cases and 11,700 prostate cancer deaths in the UK [1]. Bray et al. [2] reported 1,276,106 new global cases in 2018, and 358,989 prostate cancer deaths worldwide. In clinical practice, delineation of prostate tissue is based on manual localisation of the prostate from multi-parametric MRI. Due to the inter-reader variability, this subjective identification of prostate tissue is limited in reproducibility, time consuming and often requires clinical expertise. In routine radiological practice, accurate segmentation of the prostate could enable radiologists to more quickly demarcate the prostate gland. It can improve the determination of clinical markers such as prostate-specific antigen (PSA) density, which depends on MRI prostate volume estimation [3]. Accurate prostate segmentation is fundamental to planning MRI-guided biopsies and therapy but is challenging because of (i) the lack of a clear margin between the prostate and the surrounding anatomical structures, (ii) heterogeneous background texture, (iii) variation in size and shape of the prostate and (iv) the tissue intensity distribution [4]. Computer Aided Diagnosis (CAD) systems are used increasingly to assist clinical experts in the analysis of medical images.

Many automatic prostate segmentation methodologies have been developed in ultrasound, MRI and computed tomography images [5,6] to reduce subjectivity in delineating tissue boundaries. For example, Cuocolo et al. [7] performed a meta-analysis of machine learning approaches for diagnostic purposes using MRI. They concluded that these pipelines showed good accuracy results but need further investigation, better standardisation in design and proper reporting of the results. Comelli et al. [8] studied relevant radiomic features that were significantly correlated with the histopathological results. There are several deep learning based methods for prostate segmentation. Zhu et al. [9] used a deeply-supervised convolutional neural network (CNN) to pass the features extracted from early layers to later layers. Yu et al. [10] proposed residual connections into a volumetric CNN for 3D MR images. Guo et al. [11] proposed a deformable prostate segmentation method to unify deep feature learning with sparse patch matching instead of hand-crafted features. Liao et al. [12] used a different learning-base method, namely representation learning. In this method, a stacked independent subspace analysis (ISA) network is used to learn the effective features in a hierarchical and unsupervised way. Kohl et al. [13] used a simple generative model where there was only one discriminator and one generator. Zabihollahy et al. [14] segmented the prostate whole gland and zones from two modalities (T2W and ADC) based on the U-Net. Cheng et al. [15] presented segmentation of the whole prostate and central gland from only one modality (T2) using nested networks. Grall et al. [4] also adopted the conditional GAN architecture for prostate segmentation.

Supervised methods outperform unsupervised approaches with regards to classification and segmentation tasks. However, supervised methods need a lot of data to be annotated by experts and clinicians and this is time consuming. Developing methods to improve performance of deep learning methods on small datasets may improve performance of complex models with small datasets by incorporating suitable augmentation schemes. Data augmentation is a common method used to increase the diversity of data used for training deep models, reducing the need for collecting new data. Typical data augmentation techniques include cropping, rotation and flipping. There are several other techniques that have been used in traditional machine learning approaches for performing different tasks such as segmentation and de-noising. We propose three data augmentation schemes based on: (i) Super-pixel (SP); (ii) Gaussian Noise Addition (GNA) and (iii) Moving Mean (MM). A SP is a group of pixels sharing similar visual features such as pixel intensity, location or texture. Super-pixel based methods reduce the risk of assigning pixels to the wrong labels and also reduce the computational cost due to their strong adhesion to the object’s boundary. This method has been used in several computer vision applications [16]. Tian et al. [17] used a super-pixel based 3D graph cut algorithm to obtain the prostate surface. In their method, they considered super-pixels as the basic processing units to construct the graph, which was reported to provide an effective and robust segmentation of the prostate. GNA is a basic noise model used in information theory and image processing to mimic the effect of many random processes that occur in nature. The motivation to consider this approach is that MR images are generally affected by Rician noise [18] while for low signal intensities (Signal to Noise Ratio (SNR) < 2), the noise follows a Rice distribution and for larger signal intensities (SNR > 2) the noise distribution can be modelled as Gaussian distribution [19]. Therefore, adding Gaussian noise can be a good practice to generate varied samples. MM is also a common finite filtering method used in image processing [20] which is a calculation approach to analyse image pixels by creating a series of averages of different subsets of the full data set. This method has also been adapted in this study for data augmentation.

Multi-parametric MRI of the prostate can include: DWI (Diffusion Weighted Imaging), T1W (T1-weighted), T2W (T2-weighted), ADC (Apparent Diffusion Coefficient) and DCE (Dynamic Contrast Enhanced) [21] and here we have used the T2W, ADC and DWI modalities which are the core modalities of prostate MRI [22]. T2W sequences are part of all MRI protocols and the dominant signal intensities differentiates fluid, muscle and fat. DWI assesses the ability of water molecules to move freely within normal and diseased tissues. While DWI represents both actual diffusion values and T2 signal, ADC images represent the diffusion values of the tissue without T2 effects [21].

Generative Adversarial Networks (GANs) have shown success in generating different kinds of visual content and achieved state-of-the-art performance in many traditional and novel medical image applications such as medical image reconstruction, segmentation, detection, synthesis and classification [4,13,23]. This study aims to use U-Net and two well-known GAN based extensions as a novel approach to the segmentation of prostate tissues in multi-parametric MRI and to compare these models. In addition, alternative data augmentation schemes are proposed that could be used to compensate for the limited amount of available data for training, which require extensive data for good performances. The contributions of this work are:

- to incorporate three data augmentation schemes (super-pixel, noise addition and moving mean) to increase the amount of data in the training set;
- to train three models including U-Net, cGAN and cycleGAN with limited amount of training data and compensate it by using the augmented training data
- to compare their robustness on the publicly available prostate MRI dataset (completely unseen samples collected from different institution) and our clinically collected prostate multi-parametric MRI dataset (collected from the same institution).

Our qualitative and quantitative results show that the cGAN outperformed the other two models (U-Net and cycleGAN) which implies that the proposed detection and segmentation model has the potential to be utilised as an alternative to double reading by clinicians [24].

## 2. Methodology

### 2.1. Datasets

**Figure 1:**
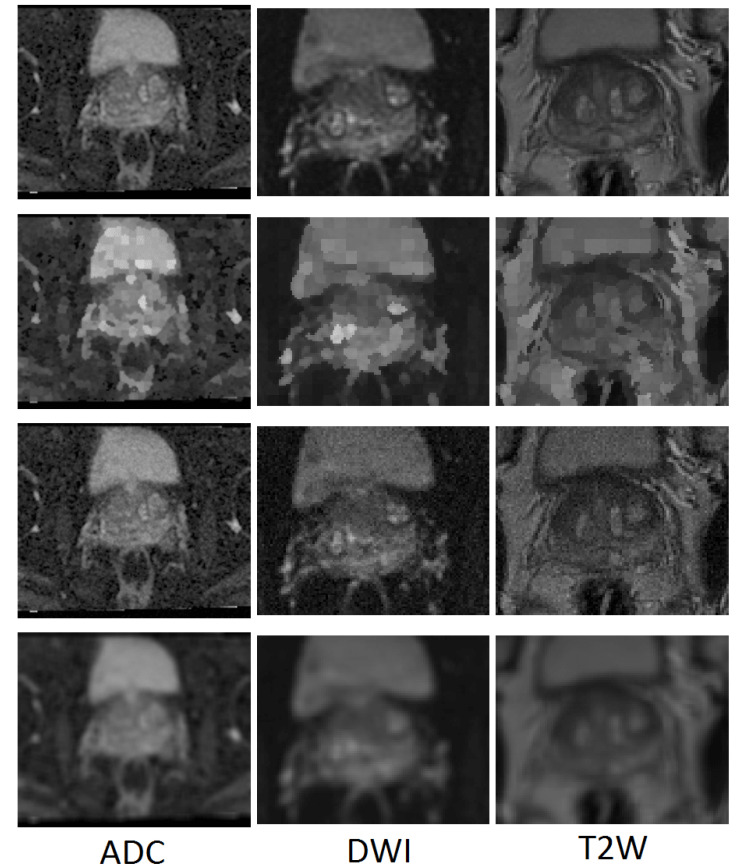
Examples of generated images for training. From top to bottom: raw image, super-pixel image, noise affected image and moving mean image.

The MR Data from 40 patients with biopsy-proven prostate cancer was collected from the Norfolk and Norwich University Hospital, UK. This dataset includes 683 ADC (Apparent Diffusion Coefficient), 683 DWI (Diffusion Weighted Imaging) and 857 T2W (T2-Weighted) MRI slices. All images were obtained with a 3 Tesla magnet GE Medical Systems, using a phased array pelvic coil, with different fields of view while covering the whole prostate and seminal vesicles in both transverse and coronal planes. The T2 small field of view sequence obtained 3.6 mm contiguous slices with a 22 × 22 cm field of view and 320 × 320 matrix. For the small field of view diffusion sequence, 3.6 mm contiguous slices were obtained with a 24 × 12 cm field of view and a 160 × 80 matrix. The MRI protocol used was the same for all the images and as such the quality was similar for the images in this cohort. There were between 16 to 32 axial slices for each case, in each modality in the dataset. All images were manually annotated by a radiologist experienced in MRI of the prostate to indicate the precise location of the prostate on a slice-by-slice basis in each modality. The radiologist segmented the prostate location on each modality separately using the Onis 2.6 Professional software (http://www.onis-viewer.com/ProductInfo.aspx?id=18). The dataset was divided into training and testing sets as 75% and 25% of the whole database based on patients, ensuring that there was no overlap between testing and training sets. For the training process, different combinations of training samples were generated by using augmented samples (covered in Section 2.2). This augmentation was done separately for each modality. The number of images created in each data augmentation step was equal to the number of raw images in that modality. To obtain objective results, the test set included only raw images. Examples of super-pixel, noisy and moving mean images are shown in Figure 1.

**Figure 2:**
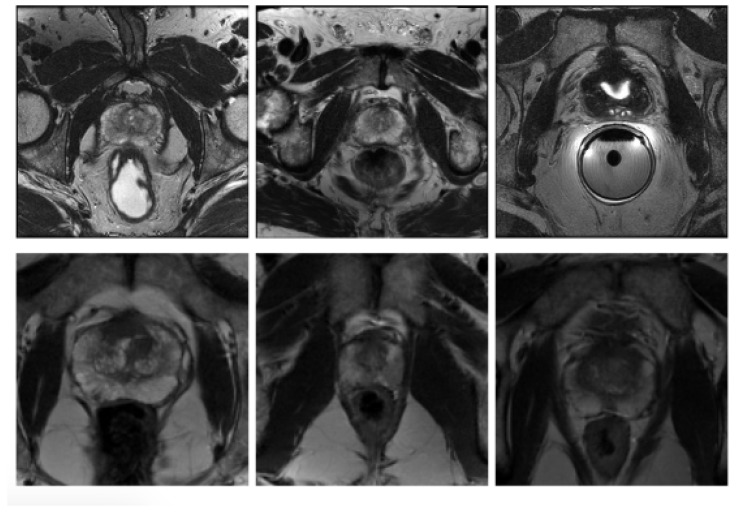
Some slices from different data cohorts to show appearance differences with T2 modality. First row: PROMISE12 dataset. Second row: our clinically collected dataset.

In order to examine the generalisability of the models on another representative set of MR images acquired in a clinical setting, the publicly available dataset (PROMISE12) [25] was used. This dataset contains 50 cases including T2-weighted MR images of the prostate. This dataset is multi-center and multi-vendor and has different acquisition protocols, with various prostate sizes and appearances. Details of the acquisition protocols for the different centers is explained by Litjens et al. [25]. Due to different MRI protocols (e.g., differences in slice thickness, magnet strength, with/without endorectal coil), the quality of the images was different in this cohort. Figure 2 shows some slices from different data cohorts to show appearance differences. There is a spread in prostate sizes and appearance in all images in our clinical cohort and the public dataset.

All images used in the testing phase were collected using different devices with different resolutions and scan protocols, while these variations were not included in the training process. We trained the models on our clinical study dataset but evaluated the performance of the models on our private clinical study dataset and the PROMISE12 public dataset. All images with different sizes were zero-padded (if needed) and scaled to 128 × 128 pixels.

### 2.2. Augmentation Schemes

#### 2.2.1. Super-Pixel (SP) Approach

Inspired by Tian et al. [17], we propose to generate super-pixels to group similar pixels in prostate MR images and use them in the training dataset. The Simple Linear Iterative Clustering (SLIC) algorithm [26] was used to cluster the relevant pixels in prostate MR images in the combined three-dimensional gray and image plane space and efficiently generate compact super-pixels. A super-pixel is defined as an image patch that is better aligned with intensity edges. These super-pixels can produce highly irregular super-pixels on MR images, with widely varying sizes and shapes. Therefore, it is of critical importance to define a suitable number of clusters. The number of segments (clusters) was tested in the range [200, 1500] and chosen to be 1200 per image. This value was selected based on the empirical evaluation in order to achieve a uniform number of segments.

#### 2.2.2. Gaussian Noise Addition (GNA) Approach

Developed by Grall et al. [4], Gaussian noise with (σ = 0.5) was added to images from all three modalities (ADC, T2W and DWI) and the resulting noisy images were used along with the raw training samples in the training step of model development.

#### 2.2.3. Moving Mean (MM) Approach

Using this approach, the raw image was divided into 5 × 5 non-overlapping grids and the average pixel value of each grid is calculated independently to create a series of averages from different subsets of the whole MR image. Then the average pixel value was assigned to all pixels of the specified grid. This procedure smooths the image and removes the sharp features in it. The grid size was selected based on the empirical evaluation to achieve good segmentation performance.

### 2.3. The Deep Learning Frameworks

#### 2.3.1. U-Net

The U-Net architecture [27] consists of contracting and expansive paths. The contracting pathway consists of a sequence of convolutions, followed by rectified linear units (ReLU) and max pooling operators, all contributing to the reduction in the spatial information and increase in the salient contextual information from images. The expansive pathway combines the feature and spatial information through a sequence of symmetrical up-convolutions, up-sampling operators and concatenations with high-resolution features to accurately localise the segmented target. The loss function is computed by performing a soft-max function over the final feature map from the expansive pathway, combined with the cross-entropy loss function, in a pixel-wise manner. We have trained this model as a benchmark model to investigate the performance of other models as described in the subsequent subsections.

#### 2.3.2. Conditional GAN (cGAN)

The first GAN architecture that was introduced by Goodfellow et al. [28], has a generator and a discriminator. The generator’s training objective is to produce realistic images from a latent space or random noise for the targeted data distribution. The images generated by the generator are evaluated by the discriminator which classifies whether the generated images are accepted or not. The generator tries to fool the discriminator while the discriminator tries to detect the generator’s falsely created images as much as possible. CGAN [29] is an extended version of GAN and the key idea behind it is to generate images that are indistinguishable from original images (desired output) using an adversarial loss. 3D prostate tissue detection and segmentation using MRI scans is implemented as finding the prostate tissue on each 2D MR scan in terms of its semantic label map representing the desired tissue in the image. This model uses paired data during the training and is regarded as a supervised methodology. It learns a mapping from conditional image *x* and *z* to output *y*, which can be demonstrated as G:x,z→y. The goal of the generator is to minimise the overall loss function by generating similar images to the target mask images, where the discriminator *D* tries to classify the generator’s created images as truly generated or not and to maximize the defined loss function stated as:

(1) $$G^{*}=arg\;min_{G}\;max_{D}\;L_{cGAN}\left(G,D\right)+\lambda L_{L1}\left(G\right).$$

where $L_{cGAN}$ can be expressed as:

(2) $$\begin{array}{c} L_{cGAN}\left(G,D\right)=E_{x,y}\left[logD\left(x,y\right)\right]+E_{x,z}[log\left(1-D\left(x,G\left(x,z\right)\right)\right]. \end{array}$$

and the generator learns to produce output images similar to ground truth (*x*) under the L1 condition:

(3) $$L_{L1}\left(G\right)=E_{x,y,z}[||y-{G\left(x,z\right)||}_{1}].$$

With regard to the network architecture, a U-Net based structure [27,29] is used for the generator and a convolutional PatchGAN classifier [29] is used for the discriminator. The PatchGAN determines if 70 × 70 overlapping image patches are correctly generated.

#### 2.3.3. CycleGAN

The CycleGan model [30] aims to learn mapping between domains without using paired data. Its architecture consists of two mapping functions *G*: *X*→*Y* and *F*: *Y*→*X*. The model aims to learn mapping functions between domains *X* and *Y* given training images {$x_{i}{\}}_{i=1}^{N}$, where $x_{i}\in X$ and {$y_{j}{\}}_{j=1}^{M}$, where $y_{j}\in Y$. The goal of the mapping function *G* is to generate images G(x) indistinguishable from images in domain *Y* and minimise the loss function in Equation (4), where the discriminator $D_{Y}$ is trying to distinguish the generator’s generated images and maximise the overall loss.

(4) $$\begin{array}{c} L_{GAN}\left(G,D_{Y},X,Y\right)=E_{y∼p_{data}\left(y\right)}\left[logD_{Y}\left(y\right)\right]+\\ E_{x∼p_{data}\left(x\right)}\left[log\left(1-D_{Y}\left(G\left(x\right)\right)\right)\right]. \end{array}$$

There is also a similar adversarial loss function for the mapping function *F*: *Y*→*X* and the discriminator $D_{X}$:

(5) $$\;min_{F}\;max_{D_{X}}\;L_{GAN}\left(F,D_{X},Y,X\right)$$

The mapping functions need to be cycle-consistent. When we map each image x from domain *X* to domain *Y*, it is brought back to the original image i.e., x→G(x)→F(G(x))≈x, which is called *forward cycle consistency*. A similar idea holds for the generator *F* and every image in domain *Y*, which is called *backward cycle consistency* i.e., y→F(y)→G(F(y))≈y. These constitute cycle consistency loss as stated in (6):

(6) $$\begin{array}{c} L_{cyc}\left(G,F\right)=E_{x∼p_{data}\left(x\right)}[||F\left(G\left(x\right)\right)-{x||}_{1}]+\\ E_{y∼p_{data}\left(y\right)}[||G\left(F\left(y\right)\right)-{y||}_{1}]. \end{array}$$

Therefore, the final loss function of the cycleGAN is:

(7) $$\begin{array}{c} L\left(G,F,D_{X},D_{Y}\right)=L_{GAN}\left(G,D_{Y},X,Y\right)+\\ L_{GAN}\left(F,D_{X},Y,X\right)+\lambda L_{cyc}\left(G,F\right). \end{array}$$

where λ indicates the importance of cycle consistency loss. In the network architecture, a ResNet [31] with 6 blocks is used for the generator and a 70 × 70 PatchGANs is used for the discriminator.

### 2.4. Training

For each MR modality used in this study (DWI, T2W and ADC), raw images were combined with the augmented images (described in Section 2.2) to create different types of datasets. Using each created dataset, cycleGAN (https://github.com/junyanz/CycleGAN) and cGAN (https://github.com/phillipi/pix2pix) models were trained. All the network training was performed within the Torch framework with a NVIDIA GeForce GTX 1080 GPU on Intel Core i7-4790 Processor. To achieve a better validation of the experiments, 4-fold cross validation was performed. The detection and segmentation performance of each trained model for the prostate tissue was evaluated only on the raw test images. All networks were trained with 70 epochs for the batch size = 1 as reported by Zhu et al. [30]. This number of epochs was observed to be sufficient for the generator’s loss function to reach a steady state in which there were no more visually perceived improvements in the predicted masks.We tried a variable range of learning rates for both models. We tested in the range [0.00001, 0.001] and empirically found that the learning rate of 0.00004 and 0.0002 worked well for the cGAN and the cycleGAN, respectively. These values were different for the U-Net. This model was trained with 150 epochs with the batch size = 1, learning rate = 0.01 using the cross-entropy loss function. The other parameters were kept as default [29,30] which included: the number of iterations at starting learning rate = 200; momentum term of adam = 0.5; the model used for the which-model-netD = basic and lambda = 100 for the cGAN model. Similar parameters were used for the cycleGAN with number of iterations at starting learning rate = 100; number of iterations to linearly decay learning rate to zero = 100; instance normalization; the model used for the which-model-netD = basic; content loss type = pixel; weight for both cycle losses (A−>B−>A and B−>A−>B) = 10; loss = least square GAN; the size of image buffer = 50 and the identity mapping = 0.5.

### 2.5. Post-Processing

**Figure 3:**
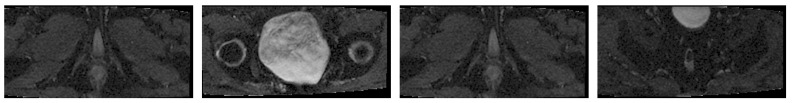
Some examples containing no prostate tissue. These slices were mostly from the top or the bottom slices in a whole 3D scan.

The prostate is a relatively small part of each 2D image in the 3D MRI volume and is surrounded by other anatomical structures such as the rectum, the bladder or the seminal vesicles and the prostate tissue is often located in the image centre, away from image borders. When detecting prostate tissue, due to the inter-patient volume variability and slice thickness differences, distinguishing the interface between the prostate and surrounding tissues was challenging. This means that it was difficult to predict true negatives where there existed no prostate tissue. This occurs at the top and bottom slices in the MRI scan where there was no prostate tissue. Most of the time, the trained models were detecting a false positive. Some of examples are shown in Figure 3. Therefore, in order to eliminate false-positive predictions, we put a constraint in the post-processing step. Detected segmentation masks which were close to the image boundary were removed and only the masks that were located between 1/4 and 3/4 of the height, and 1/4 and 3/4 of the width were maintained. Moreover, the top and bottom slices of the prostate are disregarded as they are always empty of targeted tissue. Finally, using connected component analysis, the largest object is extracted as the output.

### 2.6. Evaluation Metrics

The detection and segmentation performances was evaluated using common metrics [25]: (1) the Dice Similarity Coefficient (DSC), (2) the 95% Hausdorff distance (HD) and (3) F.Score for the unseen cases in the testing sets.

## 3. Results and Discussion

**Figure 4:**
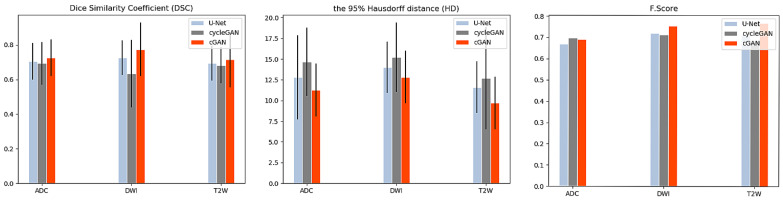
The mean and standard deviation values computed for the Dice Similarity Coefficient (DSC), Hausdorff distance (HD) and F.Score metrics using U-Net, cycleGAN and cGAN models, regarded as “base-models". Base models are trained only on the raw data. *X*-axis represents the MRI modality; *y*-axis represents the mean value and vertical black line represents the standard deviation.

**Figure 5:**
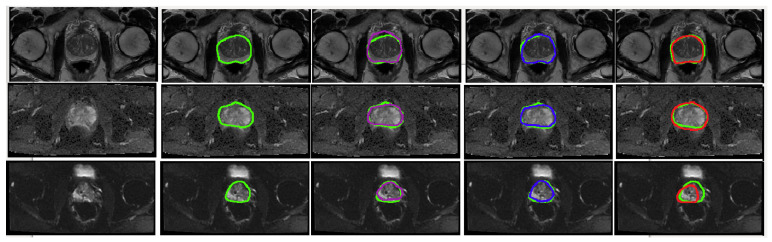
Visual representation of the predicted segmentation for each model and each modality for a single slice from a case study. From the top row to the bottom: T2W, ADC and DWI. From left to right: Raw test image, Ground truth boundaries in green, U-Net’s prediction in purple, cGAN’s prediction in blue and cycleGAN prediction in red. The quantitative results show the best performance for cGAN followed by U-Net for all evaluation metrics. Scaled images are shown in Figure A1 for better visualisation.

The segmentation performances of U-Net, cGAN and cycleGAN models were assessed in terms of DSC and HD on the base-model (the model that was trained only on the raw images); while the detection performance of these models was reported in terms of F.Score and shown in Figure 4. Based on our evaluation metrics, we conclude that the best segmentation performance in all modalities is achieved by the cGAN model, followed by the U-Net model. This out-performance of the cGAN model can be justified by comparing its structure with the U-Net architecture. The generator of the cGAN model is built on a U-Net model architecture; while its performance is adjusted by an additional discriminator (as covered in Section 2.3.2). Using the adversarial loss, the discriminator forces the generator to produce more robust and realistic output images in every iteration in contrast to the U-Net. On the other hand, comparing the performance of the U-Net and the cycleGAN model, we observed that the U-Net has better performance. Although the cycleGAN model has two discriminators, without the supervision of paired input-output data it was not able to learn robustly and segmentation performance got the lowest values for the evaluation metrics compared to the other two models. Figure 5 shows visualization results of these networks for each modality.

Table 1 shows the quantitative results in terms of mean and standard deviation of the evaluation metrics for each created dataset (see Section 2.2) and each modality. Based on our quantitative results, for the ADC modality, the best results were obtained when the model was trained on the dataset combining raw and GNA images. Training the networks with the combination of SP, MM and raw images could also improve the learning performance for this modality compared to using only the raw data. However, the nature of the noisy images became slightly different from the raw images with regards to the spatial and contrast resolution. This was more adaptive for the ADC modality in both cGAN and cycleGAN models. The captured ADC modality images were more grainy and adding more noise to them could affect the spatial and contrast resolution more than the other two modalities. This led to the reduction of the clarity and distinguishability of the interface between the prostate and surrounding tissues and more distinct images were created from the raw images. This outcome was consistent among the three evaluation metrics.

**Figure 6:**
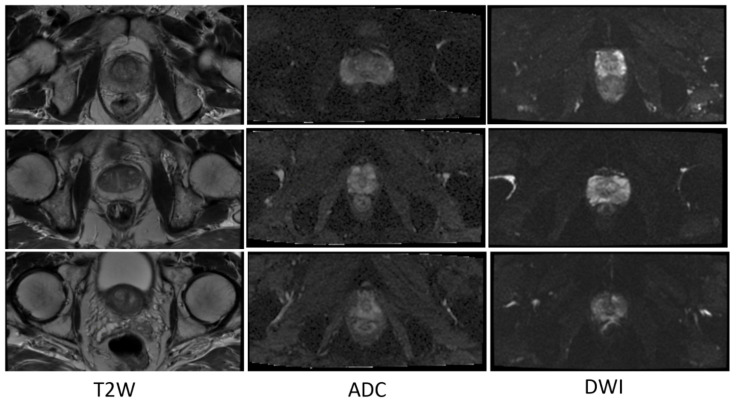
Some examples of T2W, ADC and DWI MR images from our dataset, which demonstrates that DWI and ADC modalities have lower signal around the prostate compared to the T2W modality. Scaled images are shown in Figure A2 for better visualisation.

**Figure 7:**
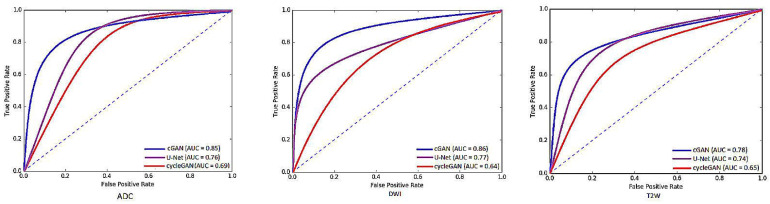
The ROC curves and AUC values for different networks used in this study applied to three modalities (ADC, DWI and T2W).

In terms of the T2W modality, similar outcome was achieved with regards to the training samples used during learning procedure for the cGAN model. The combination of raw and GNA was the best in the cGAN model; while the combination of raw and SP obtained the best output results in the cycleGAN model. To justify this by investigating the T2W modality images, we observed that the images in this modality show anatomy, especially the peri-prostatic structures, more clearly than other modalities. Figure 6 shows that ADC and DWI images have low signal around the prostate compared to the T2W modality. Superpixelizing the T2W images groups the pixels better and makes the surrounding anatomy more visible for the cycleGAN network. In the cycleGAN model there are two different domains and there is no paired input-output relation between them. The generators are penalized by the adversarial loss with the cycle consistency loss, which forces the generators to not only do the segmentation but also synthesize the original MR image from the created mask back again. As a result, the network learns a mapping between the training images and their masks and vice versa. Therefore, for the SP approach, learning is more optimised. When we compare the results of the raw plus SP images on the three modalities in the cycleGAN model, it is also consistent with this result that training using the T2W modality images obtained the best results (with DSC values of 0.748) while the DSC outcomes for DWI and ADC modalities were 0.702 and 0.710, respectively. For the DWI modality, the best results for the cGAN and cycleGAN models were achieved by using the raw plus MM images as the training set. The MM approach removes the unnecessary details from an image helping the network to focus on prostate tissue as the target part of the image during the training process. It shows consistent results with regards to the three evaluation metrics. The ROC curves and AUC values for these networks, applied to three modalities (ADC, DWI and T2W), are shown in Figure 7.

According to our quantitative results, combining the SP, GNA and MM images individually with the raw images could increase the segmentation accuracy for both cGAN and cycleGAN models. However, combining the samples all together, we concluded that it is not necessarily the number of training images that affects the learning performance but discriminative samples are more important and these discriminative samples in each modality can be generated differently based on the nature of the images and their contents. In other words, it is not just the volume of the training data but their quality and distinctiveness that will make a difference during the training. Prostate tissue and its surrounding tissue have subtle demarcation and generating distinctive samples that can be helpful for training purposes is not easy. In our study, we showed that providing similar samples will focus on these samples and therefore memorising is more feasible. For the detection performances, based on the numerical values provided in Table 1, the best results were obtained using the raw+MM in the training with the cGAN model for DWI and raw+GNA for ADC and T2W modalities, achieving the F.Score of 0.838, 0.807 and 0.804, respectively.

Comparing the cGAN and cycleGAN models, based on our results, we conclude that the cGAN model achieved better results than the cycleGAN model. Unlike the cycleGAN model, the cGAN has paired input-output images and both the generator and discriminator could see the ground-truth data; while the cycleGAN model learns to transfer images from one domain to another with lack of the supervision of paired images. To determine statistical significance of our results a paired t-test was used. The cGAN network achieved *p*-value < 0.0001 using the average DSC and average 95% HD values compared to the cyceGAN network in all modalities.

To demonstrate the clinical usability of these models and evaluate their generalisability, we tested the models with the public dataset PROMISE12. This dataset contains images captured with different scanners and protocols. This was not used for model training, since its heterogeneity was desired for model testing. The experimental results presented in Table 2 show that the trained networks were able to effectively perform automatic segmentation of prostate tissue with DSC = 0.75, 0.72 and 0.70 using cGAN, U-Net and cycleGAN, respectively which were comparable to the results gained on our clinical study dataset with DSC = 0.78, 0.76 and 0.73 using cGAN, U-Net and cycleGAN, respectively. Table 2 also compares the evaluation results of different prostate tissue segmentation methods, developed based on the recent deep learning approaches. Our trained models performed comparatively although they were trained on limited numbers of data which were artificially augmented by our proposed augmentation approaches. The use of a public dataset for external validation with such a limited loss in accuracy between the internal and external test sets is a significant advantage of our proposed techniques.

## 4. Conclusions

In recent years, generative adversarial networks have received significant attention from machine learning and computer vision communities because of their ability to generate highly realistic images with the adversarial loss created by the discriminator. This has also been used in many medical imaging problems such as classification, image reconstruction, synthesis, detection or segmentation. In this study, our contribution was to detect and correctly segment prostate tissue in different MR modalities using cGAN, cyleGAN and U-Net models. We observed the robustness and generalisation performances of such simple but effective GAN models in detection and segmentation tasks by using various data-augmentation methods including Super-pixel, Gaussian Noise Addition and Moving Mean approaches. Our qualitative and quantitative results show that the cGAN outperformed the other two models (U-Net and cycleGAN) which implies that the proposed detection and segmentation model has the potential to be utilised as an alternative to double reading by clinicians while reducing the time burden and improving patient outcomes.

## Figures and Tables

**Table 1:** Quantitative results of the segmentation/detection for each modality using cGAN and cycleGAN models.

		cGAN Model			cycleGAN Model		
**Modality**	**Training Dataset**	**DSC**	${\mathrm{HD}}_{95}$	**F.Score**	**DSC**	${\mathrm{HD}}_{95}$	**F.Score**
ADC	Raw	0.715 ± 0.106	11.24 ± 2.07	0.692	0.693 ± 0.12	14.67 ± 7.02	0.697
	Raw + GNA	**0.802 ± 0.03**	**8.35 ± 2.12**	**0.807**	**0.759 ± 0.16**	**11.97 ± 5.62**	**0.796**
	Raw + SP	0.786 ± 0.16	9.83 ± 8.11	0.759	0.710 ± 0.34	12.54 ± 6.31	0.772
	Raw + MM	0.763 ± 0.18	10.87 ± 6.80	0.782	0.735 ± 0.60	12.71 ± 6.00	0.726
	Raw+SP+MM+GNA	0.681 ± 0.25	12.56 ± 3.47	0.673	0.663 ± 0.18	14.96 ± 9.51	0.694
T2W	Raw	0.701 ± 0.24	10.69 ± 4.02	0.761	0.689 ± 0.12	12.68 ± 4.67	0.682
	Raw + GNA	**0.789 ± 0.12**	**8.59 ± 2.26**	**0.804**	0.704 ± 0.40	10.17 ± 3.67	0.701
	Raw + SP	0.720 ± 0.23	11.87 ± 5.00	0.71	**0.748 ± 0.20**	**9.66 ± 4.28**	**0.749**
	Raw + MM	0.713 ± 0.83	10.04 ± 3.84	0.642	0.713± 0.87	12.54 ± 4.19	0.719
	Raw+SP+MM+GNA	0.682 ± 0.45	11.87 ± 7.00	0.663	0.670± 0.76	13.02 ± 6.26	0.670
DWI	Raw	0.764 ± 0.25	12.82 ± 9.89	0.754	0.633 ± 0.29	15.20 ± 8.06	0.714
	Raw + GNA	0.787 ± 0.14	11.74 ± 5.22	0.795	0.729 ± 0.10	**10.18 ± 7.04**	0.730
	Raw + SP	0.765 ± 0.12	11.78 ± 8.27	0.802	0.702 ± 0.15	12.43 ± 3.96	0.766
	Raw + MM	**0.825 ± 0.24**	**9.59 ± 2.12**	**0.838**	**0.761 ± 0.17**	**10.17 ± 2.16**	**0.784**
	Raw+SP+MM+GNA	0.746 ± 0.17	11.93 ± 4.03	0.714	0.696 ± 0.13	11.16 ± 1.62	0.704

**Table 2:** The state-of-the-art prostate segmentation approaches for the MRI-T2W modality. The HD is reported in ‘mm’ except for the datasets that contain data from multiple centres. In this case, the HD is reported in pixels (shown as pix).

Reference	Deep Learning Base Methodology	Dataset	HD (mm)	DSC
Zhu et al. [9]	deeply-supervised CNN	National Cancer Institute, NIH, USA	-	0.885
Yu et al. [10]	Volumetric ConvNets	MICCAI PROMISE12	5.41	0.864
Guo et al. [11]	deep feature learning & sparse patch matching	University of Chicago Hospital	7.43 ± 2.82 pix	0.87 ± 4.0
Liao et al. [12]	stacked independent subspace analysis network	University of Chicago Hospital	8.2 ± 2.5	0.86 ± 2.2
Kohl et al. [13]	a simple generative model	National Center for Tumor Diseases, Germany	-	0.41 ± 0.28
Grall et al. [4]	conditional generative adversarial model	Norfolk&Norwich University Hospital	6.58 ± 3.00	0.73 ± 0.16
U-Net model used in this work	U-Net model	our private dataset (Section 2.1)	6.11 ± 1.49	0.76 ± 0.10
cycleGAN model used in this work	unpaired image-to-image translation(cycleGAN)	our private dataset (Section 2.1)	6.63 ± 2.94	0.73 ± 0.20
cGAN model used in this work	conditional GAN model	our private dataset (Section 2.1)	5.90 ± 3.59	0.789 ± 0.12
U-Net model used in this work	U-Net model	MICCAI PROMISE12	10.07 ± 0.95 pix	0.72 ± 0.03
cycleGAN model used in this work	unpaired image-to-image translation (cycleGAN)	MICCAI PROMISE12	11.02 ± 2.65 pix	0.70 ± 3.41
cGAN model used in this work	conditional GAN model	MICCAI PROMISE12	9.30 ± 2.6 pix	0.757 ± 2.18

**Figure A1:**
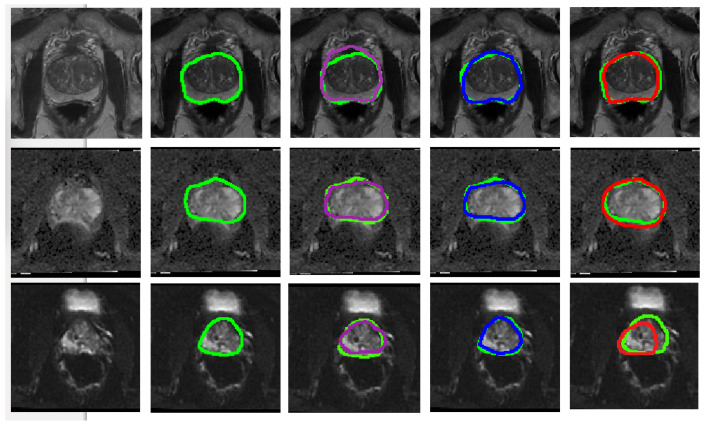
Visual representation of the predicted segmentation for each model and each modality for a single slice from a case study, where the prostate region is zoomed in and the image brightness is increased. From the top row to the bottom: T2W, ADC and DWI. From left to right: Raw test image, Ground truth boundaries in green, U-Net’s prediction in purple, cGAN’s prediction in blue and cycleGAN prediction in red. The quantitative results show the best performance for cGAN followed by U-Net for all evaluation metrics.

**Figure A2:**
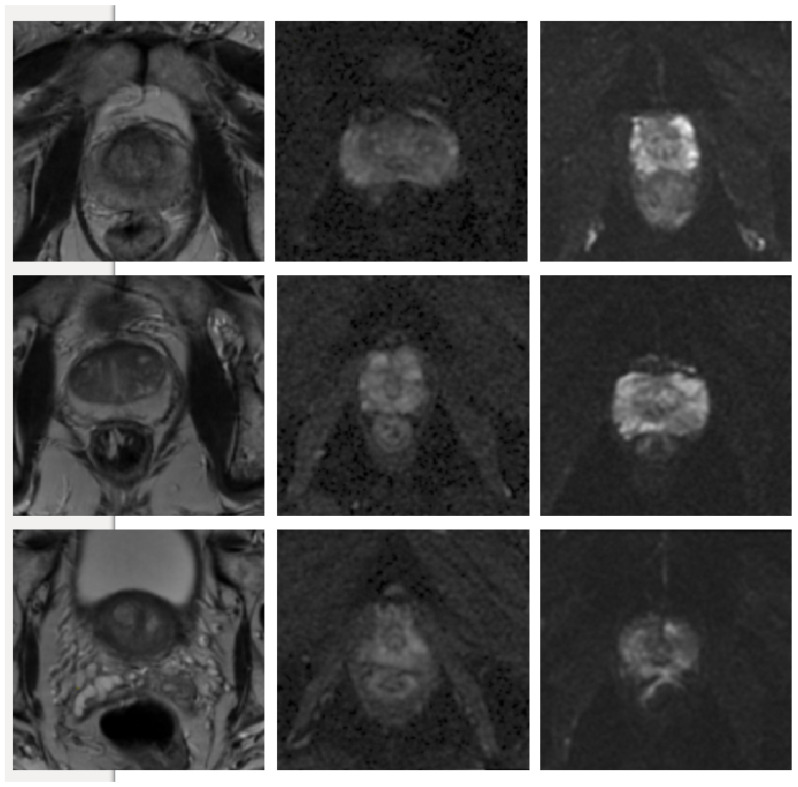
Some examples of T2W, ADC and DWI MR images from our dataset, which demonstrates that DWI and ADC modalities have lower signal around the prostate compared to the T2W modality.

## Appendix A

Figure 5 and Figure 6 from the manuscript are shown here with a smaller field of view and increased brightness for better visualisation. These images are only for display purposes and were not used during our analysis. The actual images that were used in our experiments are shown in Section 3 of the manuscript.
